# Holder Pasteurization: Comparison of Water-Bath and Dry-Tempering Devices

**DOI:** 10.3389/fped.2022.879853

**Published:** 2022-07-07

**Authors:** Katharina Müller, Luisa J. Toll, Olimpia A. Manzardo, Jana Baumgartner, Erika Nickel, Folker Wenzel, Daniel Klotz

**Affiliations:** ^1^Department of Neonatology, Center for Pediatrics, Medical Center - University of Freiburg, Faculty of Medicine, University of Freiburg, Freiburg, Germany; ^2^Faculty of Medical and Life Sciences Furtwangen University, Furtwangen, Germany

**Keywords:** human milk, pasteurization, protein, premature infant, bioactive factors

## Abstract

**Background:**

Human milk (HM) for premature infants is frequently Holder pasteurized (heated at 62.5 ± 0.5°C for 30 min) despite its detrimental effects on heat-sensitive milk components. This tolerated compromise ensures HM's microbial safety while less detrimental methods like short-time HM treatments (HTST) are still being evaluated. Dry-tempering devices (DT-HoP) were recently introduced in clinical practice due to hygienic concerns about water-based Holder pasteurizers (WB-HoP). Evidence on the impact of such dry-tempering devices on HM quality is lacking. The aim of this study was to compare protein retention rates after DT-HoP, WB-HoP and HTST.

**Methods:**

We colorimetrically determined alkaline phosphatase activity (ALP), concentrations of secretory immunoglobulin A (sIgA), and lactoferrin (LF) before and after DT-HoP, WB-HoP and HTST.

**Results:**

ALP was below the detection limit after HoP, but retained 52.8 ± 13% activity after HTST (*p* < 0.01). Secretory IgA (WB-HoP = 73.2 ± 13.5% vs. DT-HoP = 57 ± 14%, *p* = 0.0018) and LF retention (WB-HoP=47 ± 40% vs. DT-HoP=25 ± 8%, *p* = 0.07) differed between the two HoP modes. Again, retention was better maintained after HTST compared to HoP (80.4 ± 23% sIgA and 70 ± 42% LF concentration, all *p* < 0.01).

**Conclusion:**

Dry-tempering milk lowers even further the quality of HM when performing HoP compared to water-bath pasteurization, while HTST warrants continued evaluation for clinical application.

## Introduction

Human milk (HM) is the recommended type of enteral nutrition for premature infants but it is frequently pasteurized to render inactive viruses and reduce bacterial counts (1, 2). Holder pasteurization (HoP) represents the current gold standard for pasteurization whereby HM is heated to a plateau temperature of 62.5 ± 0.5°C with a holding time of 30 min. However, all heat treatments degrade a wide range of bioactive HM components, thus affecting HM quality and may therefore ultimately affect the clinical outcome of premature infants (3–5). Treatments that limit heat exposure, such as high-temperature short-time treatment (HTST), i.e., heating HM at 62–72°C for mere seconds, is therefore beneficial in retaining those bioactive components, and is currently being tested for treating human milk (6).

Recently, because of hygienic concerns and feasibility issues, solid bodies such as aluminum alloy thermostats (dry-tempering, DT) were introduced into clinical use. Those thermostats consist of electrically heated aluminum alloy blocks with customized individual boreholes for feeding bottles. Automatic processes guided by a sensor fitted reference bottle perform a Holder pasteurization whereas the milk is heated by the alloy block, foregoing any need of water as heat conductor. However, the effects of such thermostats on HM quality compared to water-bath pasteurizers have not been assessed.

We hypothesized that heat conduction while dry-tempering may be less efficient, potentially resulting in HM's longer heat exposure and an even further drop in its beneficial protein content compared to water-bath based pasteurization.

We aimed to assess the impact of water-bath based pasteurization (WB-HoP) compared to dry-tempering pasteurization (DT-HoP) on the retention rates of lactoferrin (LF), secretory immunoglobulin A concentration (sIgA), and alkaline phosphatase activity (ALP) contrasting those results to the latest HTST technology.

## Materials and Methods

### Human Milk Sampling and Preparation

**Figure 1:**
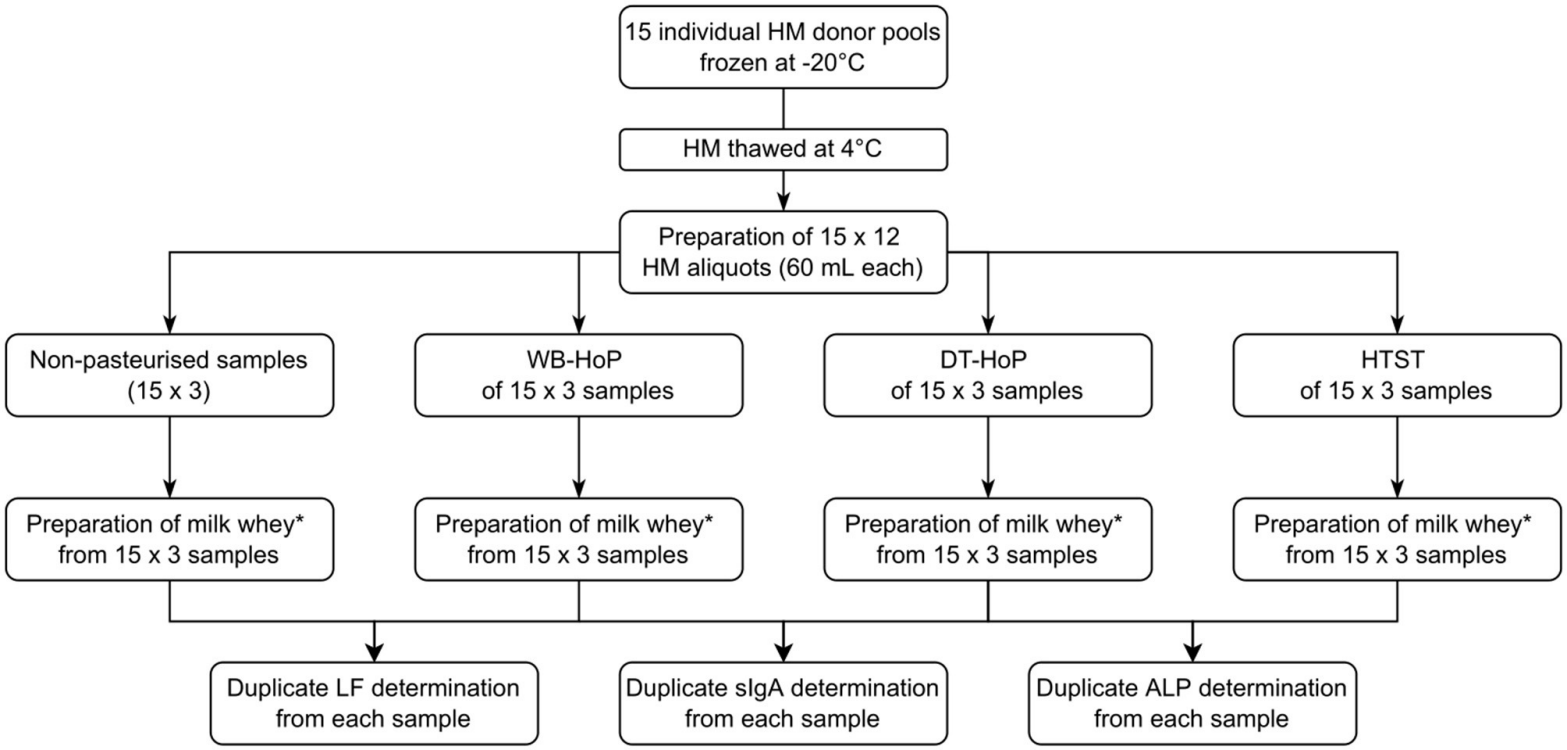
Study flowchart. HM, human milk; DT-HoP, dry-tempering Holder pasteurization; WB-HoP, water-bath pasteurization; HTST, high-temperature short-time treatment; ALP, alkaline phosphatase; sIgA, secretory immunoglobulin A; LF, lactoferrin. ^*^Preparation of milk whey: From samples 10 mL aliquots were centrifuged at 600 g for 10 min, the resulting aqueous layer was again centrifuged at 600 g for 10 min and the resulting whey supernatant was centrifuged at 2,500 g for 10 min and the resultant supernatant was then filtrated with a 0.45 μm with syringe filter.

After giving written informed consent, 15 mothers of premature infants with an excessive milk supply donated their milk for this study. HM was expressed using an electric milk pump and then stored in polypropylene bottles (Beldico, Marche-en-Famenne, BEL) at −22°C until further treatment. Mean (±SD) donation volume was 1,560 ± 124 mL per donor. Upon thawing at 4°C, overnight milk samples from each individual donor were pooled, resulting in 15 individual donor pools. We prepared 12 aliquots of 60 mL from each individual donor pool, which we then subjected to WB-HoP, DT-HoP and HTST treatment as detailed below. One aliquot from each donor pool remained untreated (Figure 1).

### Holder Pasteurization

HM samples for water-bath Holder pasteurization were heated in submerged, non-agitated bottles (SteriFeed, Medicare Colgate, UK) at 62.5 ± 0.5°C for 30 min using a HM pasteurizer (S90, Medicare Colgate) and were thereafter automatically chilled to 4°C within the device.

HM samples for dry-tempering Holder pasteurization were heated in non-agitated polypropylene feeding bottles (Beldico, BEL) using a dry-tempering device (clinitherm pasteur, MedCare Visions, Germany). The device consists of an aluminum alloy block that is heated by electric heating wires. The non-adjustable pasteurization parameters are set at 62.5 ± 0.5°C for 30 min and automatic regulation of the pasteurization process is achieved via a sensor equipped reference bottle. For temperature control toward the ambient conditions, the heating block is covered with an acryl glass insulation cover once the feeding bottles are placed in their individual boreholes. The depth and diameters of the boreholes were customized by the manufacturer according to the specific dimensions of the bottles in use. After pasteurization, the samples were chilled to 4°C in an ice bath.

### High-Temperature Short-Time Treatment

HTST treatment was performed as previously reported (Virex II, Lauf, Germany) (7). Briefly, a thin milk layer within a rotating glass flask was heated by hot air to a 62°C plateau temperature for 5 s holding time before being rapidly cooled with a cold water stream (8°C) to 32°C within the device and thereafter chilled to 4°C in an ice bath.

We carried out all pasteurization processes in a climate-controlled environment (mean ambient temperature 19.1°C, 35.2% relative humidity during the study period). Influx water to the WB-pasteurizer was temperate at 43°C. Time-temperature curves of each pasteurization cycle as measured in specific reference bottles were recorded and stored on external hard drives using device-specific propriety software. Additionally, for the purpose of this study, we recorded and stored time and temperature data from additional reference bottles using digital data loggers (176T4, Testo, GER). HM samples were stored at 4°C at all times in between later treatments.

### Human Milk Processing

From each of the pasteurized and native aliquots, 10 mL samples were centrifuged in test tubes (Falcon, BD, US) at 600 g for 10 min, the resulting aqueous layer was again centrifuged at 600 g for 10 min and the resultant whey supernatant was centrifuged at 2,500 g for 10 min. The resultant supernatant was then filtrated (Arodisc Syringe Filter 0.45 μm with Supor Membrane, Pall, US) and stored at −20°C until further analysis.

### Detection of Human Milk Proteins

Frozen filtrated whey aliquots were thawed at 4°C for biochemical analysis. We performed functional assays to determine alkaline phosphatase (ALP) activity in the samples (ALP Activity Assay Kit, Biovision, Milpitas, US). Concentrations of lactoferrin (LF) and secretory immunoglobulin A (sIgA) were measured via enzyme-linked immunosorbent assay (ELISA) kits as per the manufacturers' directions (LF: Abcam, Cambridge, UK; sIgA: Demeditec Diagnostics, Kiel, Germany).

### Data Analysis and Statistics

All pasteurization procedures were performed in triplicate, and protein determined in duplicate resulting in 6 x 15 values for each ALP, sIgA and LF from each donor. We used ANOVA, t-test or rank-sum test where appropriate for statistical analysis (GraphPadPrism V8, GraphPad, US).

## Results

**Figure 2:**
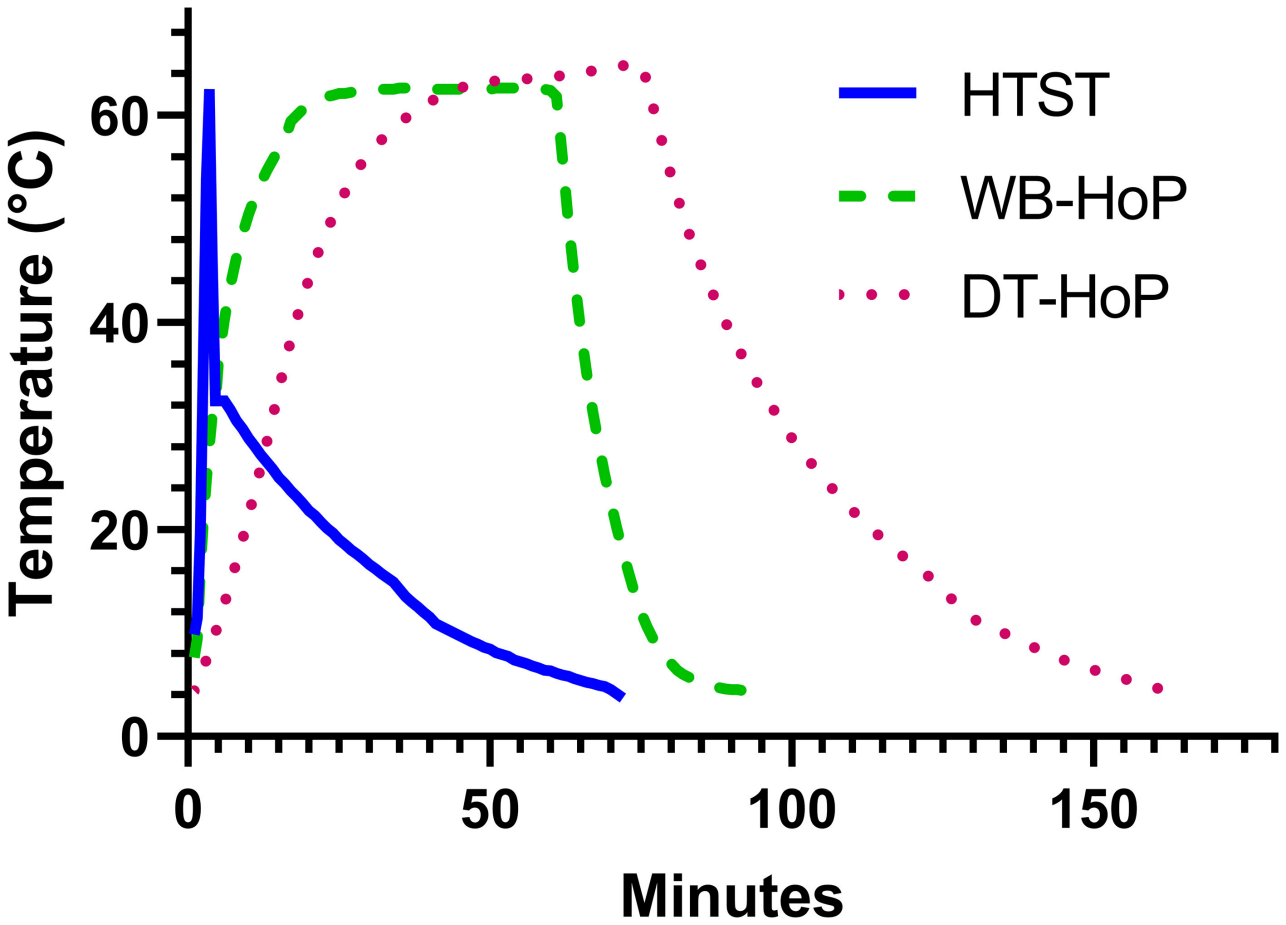
Time-temperature curves of human-milk samples during high-temperature short-time treatment (HTST, solid line), water-bath Holder pasteurization (WB-HoP, dashed line) and dry-tempering Holder pasteurization (DT-HoP, dotted line).

The time-temperature curves of the three different pasteurization processes are shown in Figure 2.

Mean (±SD) time to reach 62°C was 2.02 ± 0.2 min for HTST, to reach 62.5°C was 23.7 ± 1 min for WB-HoP and 38 ± 2 min for DT-HoP (WB-HoP vs. DT-HoP, *p* > 0.001). Time from 62.5°C to 10°C was 15.7 ± 1.2 min for WB-HoP and 43.1 ± 8 min for DT-HoP (WB-HoP vs. DT-HoP, *p* > 0.001) and time to 4°C was 39.3 ± 4 min for WB-HoP and 69 ± 14 min for DT-HoP (WB-HoP vs. DT-HoP, *p* > 0.001). Mean (SD) peak temperature was 62.7 ± 0.1°C for WB-HoP, and 64.6 ± 0.1°C for DT-HoP (WB-HoP vs. DT-HoP, *p* > 0.001).

**Figure 3:**
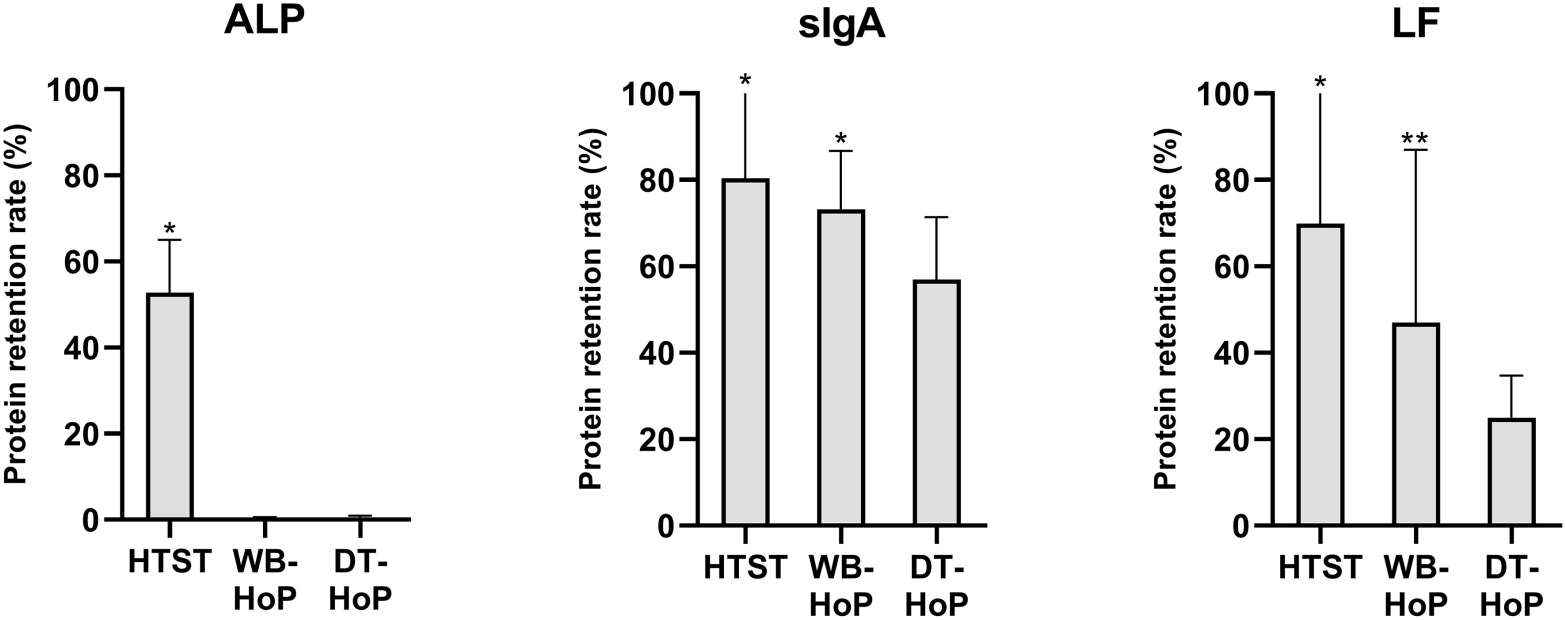
Protein retention rates for alkaline phosphatase (ALP), secretory immunoglobulin A (sIgA) and lactoferrin (LF) after thermal treatment of human milk. HTST, High-temperature short-time (pasteurization); DT-HoP, dry-tempering Holder pasteurization; WB-HoP, water-bath Holder pasteurization. *p < 0.05 (HTST vs. any HoP; WB-HoP vs. DT-HoP), **p > 0.05 (WB-HoP vs. DT HoP).

In the unpasteurized samples, mean (±SD) values were 0.68 ± 0.6 g/L for sIgA and 15.2 ± 8.6 g/L for LF concentration, ALP activity was 0.84 ± 0.3 mU/mL. Protein retention rates of ALP, sIgA and LF are illustrated in Figure 3.

In general, HTST treatment revealed higher retention rates than Holder methods. ALP activity was below the detection limit after any HoP. Otherwise, the two Holder methods' retained protein concentrations differed. sIgA concentrations were 0.47 ± 0.4 g/L after WB-HoP and 0.38 ± 0.3 g/L after DT-HoP (*p* = 0.001). LF concentrations measured 7.2 ± 6.9 g/L after WB-HoP and 3.8 ± 2.6 g/L after DT-HoP (*p* = 0.07).

## Discussion

The generally adverse effects of treating HM thermally, i.e., protein inactivation due to different degrading mechanisms, are well known (3). In this study, we demonstrated that selecting the technical application for Holder pasteurization further influences the retention rate of immunoactive HM proteins.

HoP desktop appliances that avoid traditional water-bath devices for heating milk are potentially more appealing in a clinical milk bank setting for hygienic and feasibility reasons. However, they can potentially deteriorate HM quality further, as our study shows. Both heating and cooling times differed in the tested HoP devices, as the heat-transfer performance appears to be superior in the water-bath compared to dry-tempering devices. The resulting lag times due to the diverse heat conduction displayed by the systems appear to be responsible for the differences in protein retention we observed. Furthermore, thermic inertia may also be the cause of higher peak temperatures in the dry-tempering than the water-bath device.

In line with our findings, Buffin et al. similarly compared water-bath pasteurization to air pasteurization, and demonstrated significant differences in milk's heat exposure between these two pasteurization methods, suggesting that this might significantly compromise HM's bioactive components (8).

The generally adverse effect of thermally treating HM are well described (3). Increasing temperatures resulted in higher rates of alkaline phosphatase enzyme inactivation whereas bovine milk immunoglobulins could maintain their structure resisting temperatures up to 75°C for 15 s albeit losing their antigen-binding efficacy (9, 10). Lactoferrin concentration has been consistently shown to be reduced after milk heat treatments but appears to retain its antibacterial activity even after heat treatments of 85°C for up to 10 min (11).

However, in the absence of alternative feasible treatment options, heat treatment in form of HoP is the best compromise currently available. There have been various attempts to optimize HoP. Buffin et al. demonstrated increased retention rates of IgA and LF when comparing an updated and optimized Holder pasteurizer to the same manufacturer's previous model, despite both models adhering to standard HoP criteria (12). Another modification of HoP included using a variation in the time-temperature curve as tested by Capriati et al. They applied a shortened pasteurization cycle lasting about 65 min and a peak temperature close to 72°C immediately followed by the cooling process, which revealed a trend toward better retention of triglycerides compared to standard HoP (13). By shortening the ramp time and thus heat exposure of HM proteins, Kontopodi at al. observed a tendency toward improved protein retention in milk that was rapidly preheated with an experimental HTST device before being Holder-pasteurized (14).

To preserve HM's immunologic and nutritional profile for premature infants, methods other than Holder pasteurization, such as HTST, have been evaluated. These methods were developed with the aim to achieve antiviral or antibacterial efficacy approximating the gold standard HoP, while at least weakening its detrimental impact on HM (15). Reports of increased protein retention by comparing HTST devices at 62–87°C plateau temperatures and 1–18.5 s holding times to HoP are in line with our findings (14, 16–19). Unlike those experimental continuous flow devices, we utilized a commercially available device originally designed for cytomegalovirus inactivation (7). However, this batch device appears to be less efficient in reducing bacterial counts than HoP or continuous flow HTST devices (20).

Given the available body of evidence on HTST treatment of HM, efforts should concentrate on developing non-HoP pasteurizers for actual routine use in a clinical setting (6, 15). HoP remains the best compromise currently available.

### Limitations

In actual clinical practice, the cooling process of the milk samples may not be initiated immediately after completion of the DT-HoP pasteurization thus prolonging HM heat exposure as performed in our study. In that respect, our study represents the best possible outcome after DT-HoP without an automatic cooling system, however, further HM protein degradation may occur in a real-life scenario. We did not test a dry-tempering device with an automatic cooling system that may have improved protein retention depending on the cooling process' duration. Therefore, our results cannot be generalized to apply to all DT-HoP systems; users must be aware of the pitfalls of different appliances. We only tested a limited selection of many other beneficial and heat-sensitive human-milk proteins. However, our results reveal a relevant reduction in these proteins, thus highlighting the need for further evaluation of these findings.

## Conclusion

The dry-tempering of human milk impairs the quality of HM for premature infants more than water-bath Holder pasteurization does. Universal technical standards and specifications for HM pasteurizers are lacking. Therefore, vigorous quality control measures must be carried out before adopting new appliances.

## Data Availability Statement

The original contributions presented in the study are included in the article/supplementary material, further inquiries can be directed to the corresponding author.

## Ethics Statement

This study was approved by the Ethics Committee of Albert-Ludwigs-University of Freiburg, Germany (332/19, 8th October 2019). The patients/participants provided their written informed consent to participate in this study.

## Author Contributions

KM participated in the acquisition of the HM samples and performed the pasteurization process, analyzed the data, and wrote the first draft of the manuscript. DK conceived and designed the study and helped acquire the HM samples, analyzed and interpreted the data, and contributed to drafting the manuscript. OM and JB contributed to drafting the manuscript. FW, LT, and EN designed the study, conducted the biochemical analysis, and contributed to drafting the manuscript. All authors reviewed the manuscript. All authors contributed to the article and approved the submitted version.

## Conflict of Interest

The authors declare that the research was conducted in the absence of any commercial or financial relationships that could be construed as a potential conflict of interest.

## Publisher's Note

All claims expressed in this article are solely those of the authors and do not necessarily represent those of their affiliated organizations, or those of the publisher, the editors and the reviewers. Any product that may be evaluated in this article, or claim that may be made by its manufacturer, is not guaranteed or endorsed by the publisher.
